# Angiotensin-Converting Enzyme 2 (ACE2) in the Pathogenesis of ARDS in COVID-19

**DOI:** 10.3389/fimmu.2021.732690

**Published:** 2021-12-22

**Authors:** Keiji Kuba, Tomokazu Yamaguchi, Josef M. Penninger

**Affiliations:** ^1^ Department of Biochemistry and Metabolic Science, Akita University Graduate School of Medicine, Akita, Japan; ^2^ Institute of Molecular Biotechnology Austria (IMBA), Vienna, Austria; ^3^ Department of Medical Genetics, Life Sciences Institute, University of British Columbia, Vancouver, BC, Canada

**Keywords:** ACE2, renin – angiotensin – aldosterone system, SARS-CoV-2, ARDS, acute lung injuries

## Abstract

Seventeen years after the epidemic of SARS coronavirus, a novel coronavirus SARS-CoV-2-emerged resulting in an unprecedented pandemic. Angiotensin-converting enzyme 2 (ACE2) is an essential receptor for cell entry of SARS-CoV-2 as well as the SARS coronavirus. Despite many similarities to SARS coronavirus, SARS-CoV-2 exhibits a higher affinity to ACE2 and shows higher infectivity and transmissibility, resulting in explosive increase of infected people and COVID-19 patients. Emergence of the variants harboring mutations in the receptor-binding domain of the Spike protein has drawn critical attention to the interaction between ACE2 and Spike and the efficacies of vaccines and neutralizing antibodies. ACE2 is a carboxypeptidase which degrades angiotensin II, B1-bradykinin, or apelin, and thereby is a critical regulator of cardiovascular physiology and pathology. In addition, the enzymatic activity of ACE2 is protective against acute respiratory distress syndrome (ARDS) caused by viral and non-viral pneumonias, aspiration, or sepsis. Upon infection, both SARS-CoV-2 and SARS coronaviruses downregulates ACE2 expression, likely associated with the pathogenesis of ARDS. Thus, ACE2 is not only the SARS-CoV-2 receptor but might also play an important role in multiple aspects of COVID-19 pathogenesis and possibly post-COVID-19 syndromes. Soluble forms of recombinant ACE2 are currently utilized as a pan-variant decoy to neutralize SARS-CoV-2 and a supplementation of ACE2 carboxypeptidase activity. Here, we review the role of ACE2 in the pathology of ARDS in COVID-19 and the potential application of recombinant ACE2 protein for treating COVID-19.

## Introduction

ACE2 is a homologous molecule of angiotensin converting enzyme (ACE) and functions as an enzyme (carboxypeptidase) that degrades angiotensin II peptides on the cell membrane surface (1). *In vivo*, the peptidase activity of ACE2 has been shown to suppress the renin-angiotensin system (RAS system) to improve cardiovascular or kidney diseases as well as acute respiratory distress syndrome (ARDS) (2–4). Infection with the 2003 SARS coronavirus rapidly caused severe lung inflammation in most cases and a high rate of ARDS, which made it relatively easy to identify infected individuals. On the other hand, the current SARS-CoV-2 pandemic virus is highly infectious with wide range of symptoms from asymptomatic and mild cases to some moderate and severe cases (5). It is thus difficult to isolate and contain SARS-CoV-2 infections without extensive testing strategies. There are still many unresolved issues to fully understand the molecular mechanisms of SARS-CoV-2 infections and the onset and aggravation of COVID-19.

## ACE2 As a SARS-CoV-2 Receptor

SARS-CoV-2 is an enveloped single-stranded RNA virus that belongs to the family of beta coronaviruses (6). The SARS coronavirus in 2003 is structurally and genetically highly similar to SARS-CoV-2, both of which carry a transmembrane Spike protein on the surface of the infectious virus particles. The binding of Spike protein to ACE2 on the host cell surface leads to entry of the virus into cells, a process that is required to establish the infection (7). The Spike protein of SARS-CoV-2 has a higher affinity for the human ACE2 protein as compared to the Spike of the first 2003 SARS coronavirus. The origin of SARS-CoV-2 remains unknown, but a coronavirus called RaTG13, which shows a high homology of 96% or more in its genome sequence, has been isolated from bats. It has been therefore suggested that SARS-CoV-2 might have emerged and evolved in bats (8).

**Figure 1:**
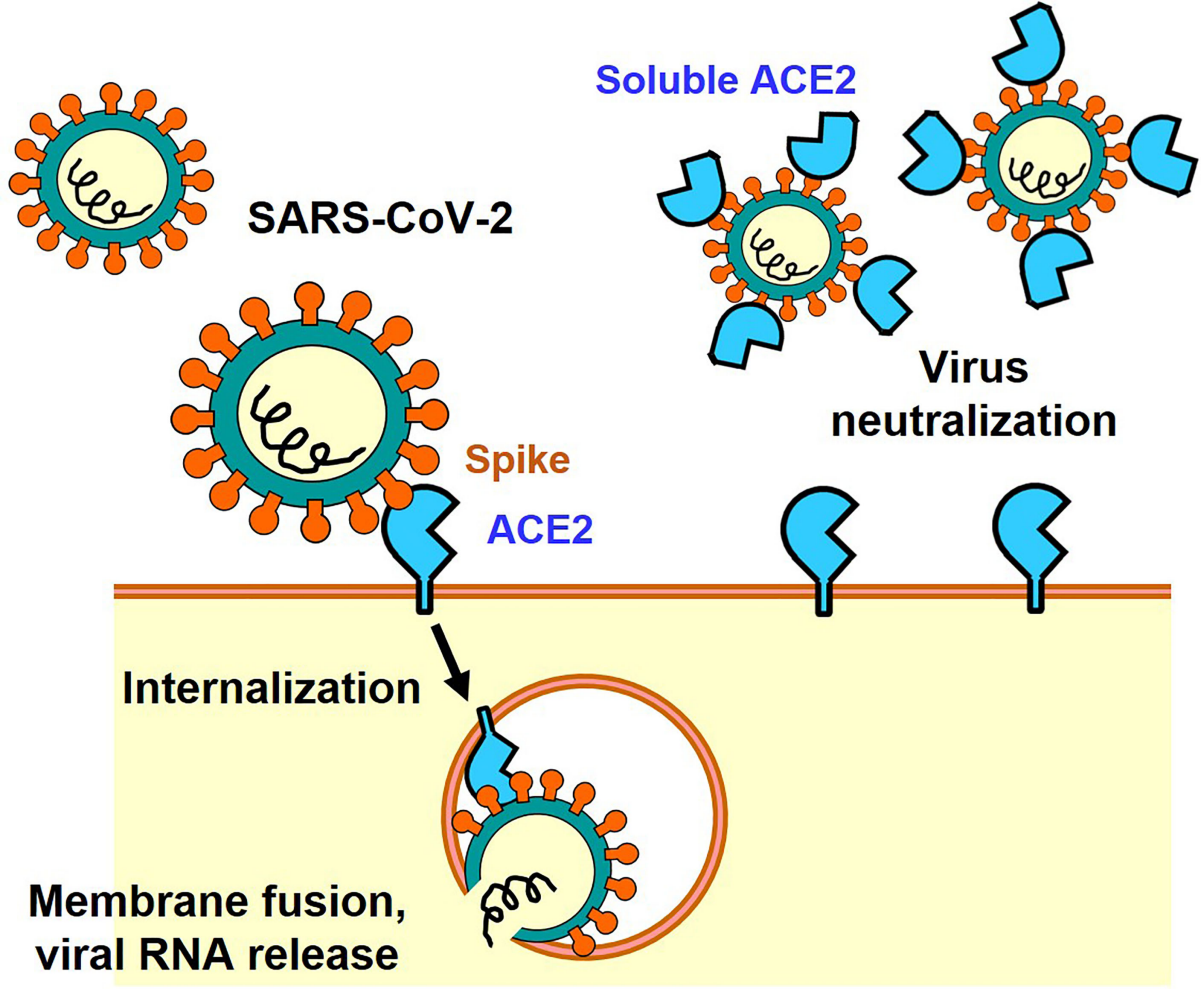
ACE2-mediated cell entry of SARS-CoV-2 and inhibition of virus infection by recombinant soluble ACE2 protein.

The Spike protein consists of an S1 region containing the receptor binding domain (RBD) that binds to ACE2 and an S2 region that promotes fusion of the viral membrane with the host cell membrane (9). Membrane fusion between host cells and the infectious virus particles is activated through cleavage of the Spike protein between S1 and S2 by host cell proteases such as transmembrane protease serine 2 (TMPRSS2) or cathepsin (7, 10). There are two modes of infection: the cell surface-mediated pathway and the late endosome-mediated pathway. Cleavage of S protein by TMPRSS2 is important on the cell surface. Furin, which is highly expressed in the cardiovascular system, induces the cleavage of S1/S2 in Spike, which might contribute to the different manifestations of COVID-19 pathologies as compared to 2003 SARS (7, 10). Consequently, it has been shown in infection experiments using cultured cells that serine protease inhibitors such as Nafamostat mesylate and Camostat mesylate suppress the intracellular invasion of SARS-CoV-2, and clinical trials are currently being conducted (2). In addition, it has been shown that structural changes in the Spike protein can also be mediated by neutrophils proteases such as elastase, one mechanism by which inflammatory cells could affect SARS-CoV-2 infections (11, 12). In the late endosome-mediated pathway, after the virus is internalized together with ACE2, structural changes in the Spike protein are induced by proteases such as cathepsin as the pH decreases in the endosome (13). Other SARS-CoV-2 receptors, such as CD147 or Lectins have also been reported, but in the absence of these additional receptors the ACE2-expressing cells are still permissive for SARS-CoV-2 (14–16). Recently, neuropilin-1 has been reported as a co-receptor for SARS-CoV-2, which facilitates virus entry through ACE2 receptor (17). Thus, ACE2 is the only essential receptor for SARS-CoV-2 infection. Irrespective if these additional SARS-CoV2 receptors might contribute to *in vivo* infections, the soluble ACE2 recombinant protein can be utilized as a molecular decoy that neutralizes the virus and thereby strongly suppresses cellular entry of the virus and thereby reduces the infection (18) (**Figure 1**). Clinical trials are currently underway to use soluble ACE2 as an antiviral drug that might be effective against essentially all SARS-CoV2 variants of concern. In addition, ACE2 functions as an enzyme that degrades peptides. Importantly, the ACE2 binding site of Spike is separated from the catalytic active site, and the binding of Spike protein does not apparently affect the enzyme activity of ACE2 recombinant protein *per se in vitro* enzymatic assays and also *in vivo* in a patient with COVID-19 (19).

## Pulmonary ACE2 Expression and SARS-CoV/SARS-CoV-2 Infections

As for the 2003 SARS coronavirus, *in vitro* biochemical analysis elucidated that ACE2 is a candidate receptor for SARS-CoV Spike protein, though multiple additional receptors were proposed. We therefore conducted the first *in vivo* SARS-CoV infections using ACE2-deficient mice, proving the first definitive proof that ACE2 is an indispensable receptor for SARS coronavirus infections *in vivo*. At the same time, we found that ACE2 is highly expressed in the lung and that the expression of ACE2 is suppressed in the lungs of SARS infected mice (20). Post-translational regulation such as intracellular localization in late endosome-mediated viral infection (**Figure 1**) or post-transcriptional regulation *via* induction of microRNA expression by NF-kB activation have been reported as molecular mechanisms of pulmonary ACE2 expression (21). Recently, we have found that SARS-CoV-2 infected hamster lungs also exhibit reduced ACE2 protein expression (22). Therefore, it is presumed that the expression of ACE2 protein is also decreased in the lung tissue of COVID-19 pneumonia patients, and it is possible that the ACE2 and RAS systems are involved in the pathophysiology of COVID-19.

## Enzyme Activity of ACE2 and Its Role in the Cardiovascular System

**Figure 2:**
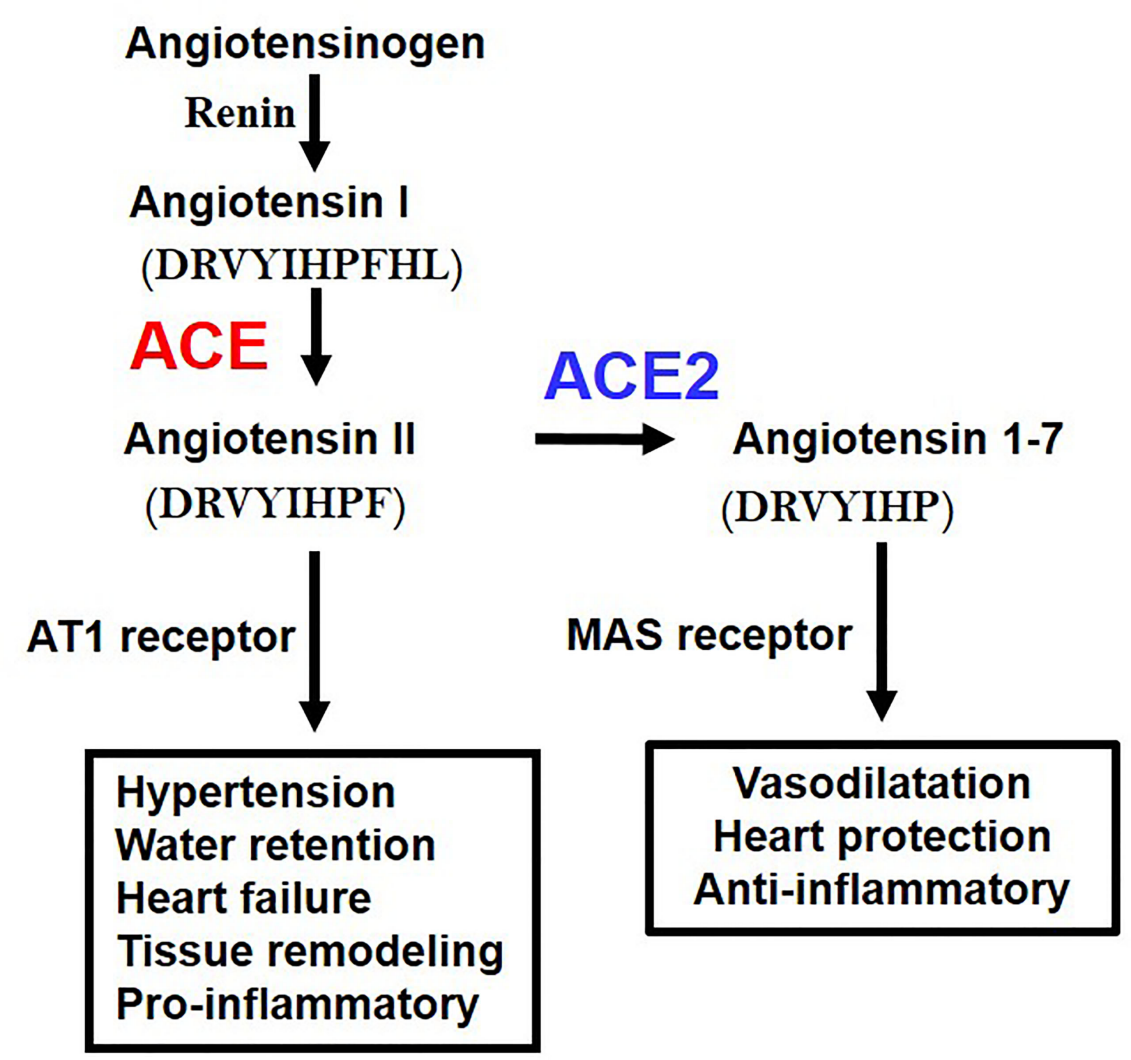
Schematic of the Renin-angiotensin system (RAS) and the central roles of ACE and ACE2. AT1 receptor, Angiotensin type 1 receptor; MAS, MAS1 Proto-Oncogene, G Protein-Coupled Receptor. The amino acid sequences of Angiotensin 1 (Ang I), Angiotensin II (Ang II), and Angiotenin 1-7 (Ang 1-7) are indicated.

While ACE2 is a receptor for SARS-CoV2, it was originally discovered as an enzyme that degrades angiotensin II (Ang II). The RAS system contributes to regulation of blood pressure, renal function, water homeostasis, electrolyte balance, or inflammation *in vivo* through the production of the vasoactive octapeptide Ang II. ACE has a metalloprotease structure in which zinc (Zn) is coordinated, thereby cleaving two amino acids at the C-terminal of angiotensin I to produce active Ang II. In contrast, ACE2 has a similar metalloprotease structure, but *in vivo* primarily cleaves Ang II as a substrate to produce angiotensin 1-7 (Ang 1-7) (23) (**Figure 2**). Whereas the ACE gene maps to the autosomal chromosome 17 of humans, we initially mapped ACE2 to the X chromosome, in multiple species. Of note, we initially cloned ACE2 in our laboratory in a fly screen for heart tube development and then made the first ACE2 mutant mice (24) that allowed us to perform the above in experiments to define the essential *in vivo* role of ACE2 in the RAS. ACE2 degrades not only Ang II but also peptides such as Apelin (APJ receptor agonist) or des-Arg9-Bradykinin (1-8) (B1 receptor selective agonist), expanding the role of ACE2 beyond the RAS (23).

Angiotensinogen, the progenitor of the Angiotensin I and Angiotensin II peptides, Renin, ACE, Angiotensin II, and Angiotensin type 1 receptor (AT1 receptor) increase the blood pressure as a positive regulatory pathway of the RAS. Mice deficient in all the above coding genes show a marked decrease in blood pressure. On the other hand, ACE2 was initially thought to have an antihypertensive effect because it decomposes Ang II, and it was expected that blood pressure would increase in ACE2-deficient mice, but intriguingly, no major abnormality was observed in blood pressure. On the other hand, it was found that ACE2-deficient mice showed progressive deterioration of cardiac function with aging, and in the pathological model of heart failure and renal failure, it was found that the genetic inactivation of ACE2 resulted in exacerbated pathologies driven by Ang II. Accordingly, ACE2 maintains the homeostasis of the circulatory system by negatively regulating the RAS, maintains cardiac contractility and suppresses tissue remodeling such as in diabetic kidney fibrosis of liver fibrotic models (4). In addition, Ang 1-7 metabolized from Ang II by ACE2 exerts functions such as vasodilation and improvement of cardiac function through activation of the Ang 1-7/MAS receptor pathway. Thus, in addition to downregulation of Ang II levels, ACE2 has multiple beneficial effects on cardiovascular pathologies, diabetic injury, fibrosis, inflammation and, most importantly for this review, acute lung injury (2–4).

## Role of RAS/ACE2 in the Lung Injury of COVID-19

**Figure 3:**
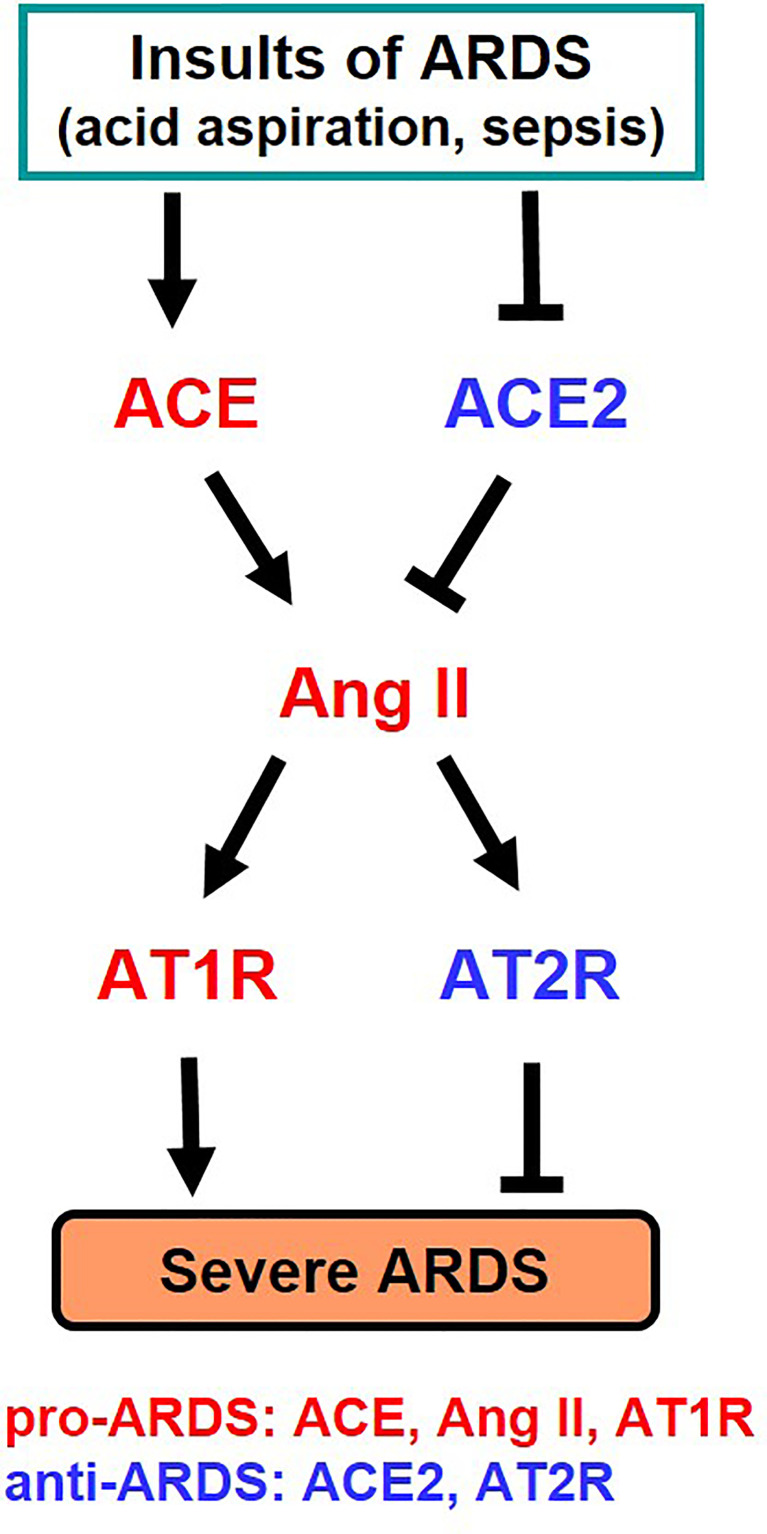
Summary of genetic study of the roles of RAS components in severe ARDS in mice. Ang II, Angiotensin II; AT1R, Angiotensin type 1 receptor; AT2R, Angiotensin type 2 receptor.

It has long been known that the activity and expression of ACE is extremely high in the lung, and analysis for clinical samples of ARDS patients had shown that the higher the expression of ACE in the *ACE I/D* gene polymorphism, the more severe the onset and severity of ARDS (25). Our study in the early 2000s, where we developed pulmonary intensive care units for mice, revealed that ACE2 has a lung protective effect in ARDS in the acute phase (26). In the ARDS/acute lung injury model of mice, ACE2-deficient mice develop markedly worsened respiratory functions, increased vascular permeability, marked pulmonary edema, neutrophil infiltration, and destruction of alveolar structures as compared to wild-type mice (26). Furthermore, administration of recombinant soluble ACE2 protein to mice with lung injury markedly improved the symptoms, that is, ACE2 functions as a lung protective factor in ARDS. On the other hand, administration of AT1 receptor blocker (ARB) or introduction of ACE gene deficiency into ACE2-deficient mice improved severe lung injury and suppressed vascular permeability. Furthermore, improvement of acute lung injury was observed in ACE-deficient mice and AT1-receptor-deficient mice (26). Deficiency of angiotensin type 2 receptor (AT2 receptor) exacerbated acute lung injury. Therefore, these genetic mapping studies in mice showed that ACE, Ang II and AT1 receptors exacerbate in acute lung injury, whereas ACE2 and AT2 receptors function as lung protecting factors (26) (**Figure 3**).

**Figure 4:**
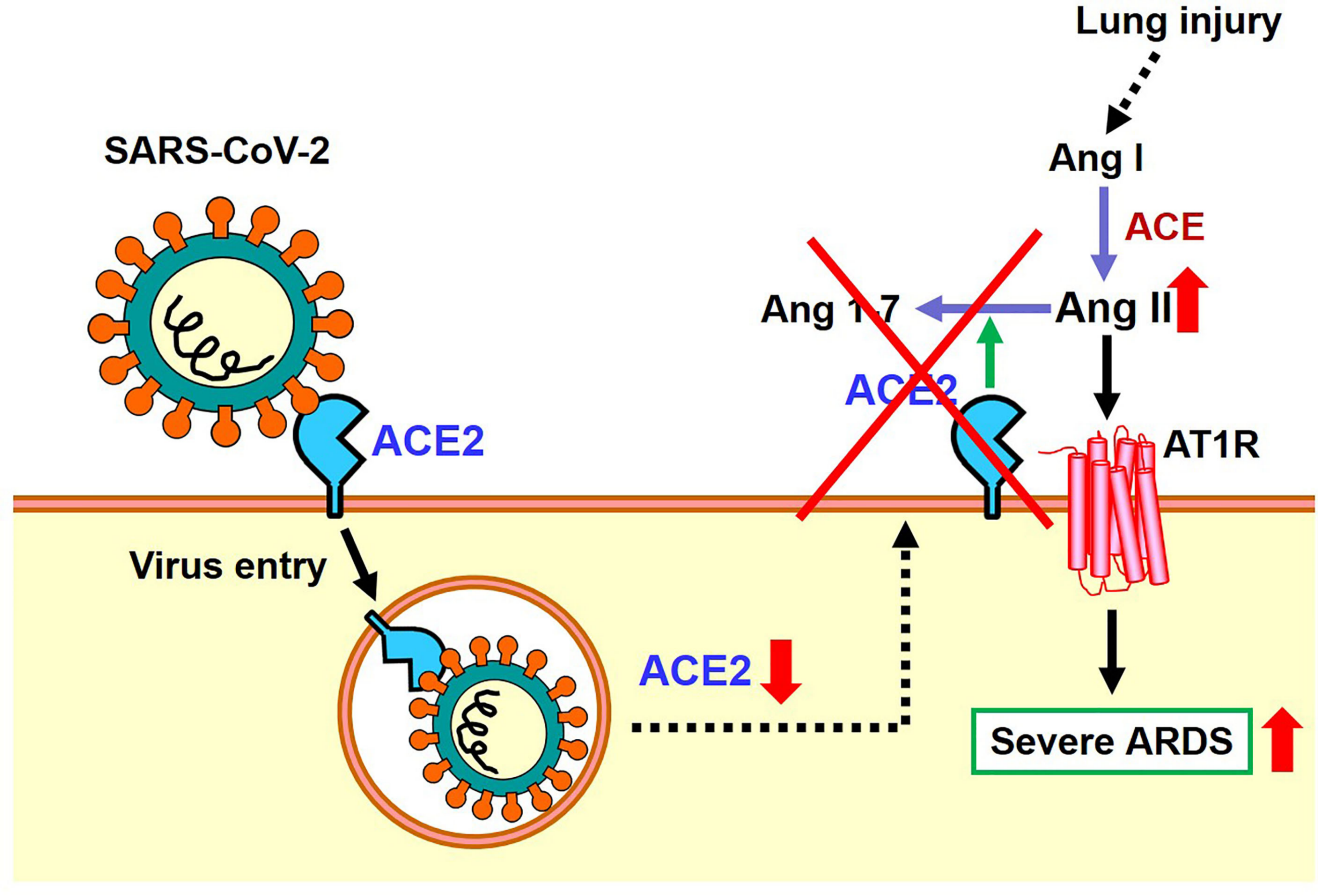
Proposed role of the RAS and ACE2 in SARS-CoV-2 infection and subsequent pathogenesis of ARDS.

As mentioned above, the expression of ACE2 protein is down-regulated in the lungs of SARS-CoV2 infected hamsters, possibly *via* internalization of ACE2 together with the attached virus, though other mechanisms might be operational. In 2003, it was found that recombinant Spike protein of the 2003 SARS coronavirus alone decreased the expression level of ACE2 on the cell surface of cultured cells. Accordingly, in the hydrochloric acid (HCl) induced ARDS model of mice, administration of recombinant Spike protein of SARS coronavirus markedly worsened respiratory functions, inflammation, and pulmonary edemas. In mice treated with Spike protein, decreased expression of ACE2 and increased Ang II levels were observed, and administration of ARB improved severe acute lung injury caused by Spike of the 2013 SARS-CoV (20). More recently, we administered SARS-CoV-2 Spike protein to a hamster with HCl inhalation-induced ARDS, which showed a similar exacerbation of acute lung injury (22). Treatment with the ACE2-like enzyme B38-CAP improved severe lung injury (22, 27). Of note, B38-CAP does not bind the SARS-CoV2 virus but carries ACE2 catalytic activity. Furthermore, we found that even in actual SARS-CoV2 infections, the pathophysiology of ARDS/acute lung injury can be improved by supplementing the enzymatic activity of ACE2 (22). These data are in line with recent data showing that soluble ACE2 can improve SARS-CoV2-induced lung injury in animal models (28, 29). Based on these results, it appears that both SARS coronavirus and SARS-CoV-2 infections activate the local RAS in the lung and downregulate ACE2 in the lungs, thereby contributing to more severe ARDS (**Figure 4**).

## RAS Inhibitors and Regulation of ACE2 Expression

From the beginning of the COVID-19 outbreak, clinical studies reported that cardiovascular diseases such as hypertension were important risk factors for aggravation of COVID-19. Interestingly, ACE2 is also a critical regulator of cardiovascular pathophysiology. After ACE2 was first discovered in 2000, our group generated first ACE2 mutant animals to explore its biological *in vivo* function, resulting in a cardiovascular phenotype (24). For example, spontaneously hypertensive rats develop heart failure due to prolonged hypertension, but the expression of ACE2 in heart and vascular tissues decreases as the disease progresses. On the other hand, it was reported that administration of ARB or ACE inhibitor (ACEi) restored and increased the expression of ACE2 in tissues by lowering blood pressure and improving the pathophysiology of heart failure (30). Similar elevations in ACE2 expression have also been observed in the kidneys of ARB-treated diabetic nephropathy model mice (31). Based on these findings, it was proposed that ARBs and ACEi prescribed as antihypertensive drugs may increase the expression of ACE2 in hypertensive patients and increase the risk of SARS-CoV-2 infection, proposing that these antihypertensive treatments might need to be changed. However, in these previous papers it has been reported that ARB and ACEi actually do not increase the expression of ACE2, but that the expression of ACE2 is normalized as the disease condition improves. Subsequent large-scale clinical studies reported that taking ARB or ACEi did not affect either SARS-CoV-2 infection or the frequency of COVID-19 onset (32). More recent data analysis of 2 million people reported that taking ARBs and ACEi rather suppressed the aggravation of COVID-19 (33), supporting our first genetic mapping studies that ACE2 positively affects prevention from the pathology of ARDS.

## Perspectives

It has recently been reported in clinical trials that the AT2 receptor small molecule agonist C21 improved respiratory failure in COVID-19 patients (34). The AT2 receptor is also called an organ-protective RAS system, and exhibits an action opposite to that of the canonical RAS system with activation of the AT1 receptor. Moreover, multiple groups are testing soluble forms of ACE2 as treatment of COVID-19, with ACE2 playing a dual role as Spike receptor and a protector of multiple organs involved in the COVID-19 pathology. We eagerly await the outcomes of these clinical studies.

## Author Contributions

KK, TY, and JMP wrote the manuscript. All authors contributed to the article and approved the submitted version.

## Funding

KK is supported by the Kaken [20H03426, 20K21566] from Japanese Ministry of Science and the Naito Foundation. TY is supported by the Kaken [20K07285] from Japanese Ministry of Science and the Takeda Science Foundation.

## Conflict of Interest

Author JMP is a shareholder of Apeiron Biologics which is developing soluble ACE2 (APN01) for COVID-19 therapy.

The remaining authors declare that the research was conducted in the absence of any commercial or financial relationships that could be construed as a potential conflict of interest.

## Publisher’s Note

All claims expressed in this article are solely those of the authors and do not necessarily represent those of their affiliated organizations, or those of the publisher, the editors and the reviewers. Any product that may be evaluated in this article, or claim that may be made by its manufacturer, is not guaranteed or endorsed by the publisher.
